# The views of GPs about using sit–stand desks: an observational study

**DOI:** 10.3399/BJGPO.2021.0203

**Published:** 2022-06-15

**Authors:** Gregory J H Biddle, Nicholas Thomas, Charlotte L Edwardson, Stacy A Clemes, Amanda J Daley

**Affiliations:** 1 School of Sport, Exercise and Health Science, Loughborough University, Loughborough, UK; 2 Clinical Innovation and Research Centre, Royal College of General Practitioners, London, UK; 3 Diabetes Research Centre, University of Leicester, Leicester, UK

**Keywords:** General practice, general practitioners, sedentary behaviour, sit-stand desks, occupational health

## Abstract

**Background:**

Occupational sitting is associated with negative health outcomes. Sit–stand workstations have been shown to reduce sitting time in office workers, although there is no evidence on whether this change to practice would be acceptable to GPs.

**Aim:**

To investigate GPs views about the use of sit–stand desks within general practice and the potential impact they may have on the nature and quality of consultations with adult patients.

**Design & setting:**

Observational study involving GPs located across the UK.

**Method:**

An online survey was emailed to members of the Royal College of General Practitioners (RCGP) and shared on social media. Only GPs working in the UK were eligible. The survey included questions on socio-demographics, GPs views about the use of sit–stand desks within their work, their levels of physical activity, total time spent sitting at work each day, and time spent at work.

**Results:**

14 142 surveys were sent by the RCGP to their members with 810 GPs responding, with a further 33 responding via social media. 60.6% of GPs would like a sit–stand desks in their consultation room, while 19.2% already had one. Most GPs thought sit–stand desks could be used for telephone consultations (91.9%) and administration tasks (92.3%). There was less agreement about whether they could be used during face-to-face consultations (35.0% agreed), with the potential impact on the doctor–patient relationship raised as the primary concern.

**Conclusion:**

The implementation of sit–stand desks had support from GPs, but their possible impact on the doctor–patient relationship should be considered in future research.

## How this fits in

The majority of GPs in the UK are likely to sit for long periods of their working day. There is no evidence about whether sit–stand desks could be used to reduce the time GPs spend sitting, leading to potential improvements in their health and well-being. Standing may also facilitate GPs conversations with patients about reducing their sitting time and increasing participation in physical activity. This study found that GPs would support the implementation of sit–stand desks within general practice, as long as consideration was given to the impact they may have on the doctor–patient relationship.

## Introduction

Sedentary behaviour in occupational settings has been associated with an increased risk of disease and conditions, including type 2 diabetes,^1^ musculoskeletal disorders,^2^ poor mental health,^3,4^ and a lower quality of life.^5^ It has been well documented that office-based employees spend a large amount of their working day sitting,^6,7^ yet data on other occupations and settings have been limited. One example of this is healthcare workers, particularly GPs, who are likely to be seated much of the day. High levels of sedentary behaviour are not only associated with poor health outcomes, but breaking up prolonged sitting with regular breaks of standing or light movement can improve markers of cardiometabolic health,^8–10^ fatigue and vigour,^11,12^ and can reduce musculoskeletal complaints.^13^

Unlike other healthcare workers, GPs' work can be centred around spending a lot of time in one place, the consultation room. This working environment has removed the need to move regularly. Therefore, it is important to explore ways that GPs might be provided with opportunities to be less sedentary at work, potentially using sit–stand desks, which allow the user to either sit or stand at work. Not only may this approach enable GPs to become less sedentary, but the use of sit–stand desks may also prompt GPs to have conversations with patients routinely about reducing the time spent sitting and increasing physical activity levels to improve health. This is important because a large part of the population typically sits for most of the day and is insufficiently physically active each day or week.^14^ Furthermore, evidence has indicated that GPs do not regularly discuss reducing sitting time and increasing participation in physical activity with patients.^15^

Doctors standing to deliver health care is not new; in hospital ward rounds, interactions are typically conducted with doctors standing and patients sitting. GP consultations, however, are typically conducted with both the patient and GP sitting. Despite evidence from non-healthcare settings that has shown sit–stand desks to be effective in reducing the amount of time employees spend sitting, introducing these in GP consultations would be a large personal, cultural, and environmental change for patients and GPs alike. There have been recent calls for sit–stand desks to be routinely implemented in general practice but, to date, there has been no evidence to support their use.^16^

Before implementing sit–stand desks within general practice, it is important to consider the views of GPs about doing so, to consider how feasible and acceptable this change to practice may be, as well as understanding potential barriers to the successful implementation. This study aimed to investigate GPs views about the potential use of sit–stand desks within general practice, their potential impact on the nature and quality of consultations with patients (adults), and the impact that GPs perceive sit–stand desks would have on their productivity and their own health. This study also aimed to explore any perceived potential barriers to successfully implementing sit–stand desks within general practice.

## Method

### Study design

This survey study was conducted in collaboration with the RCGP. Questionnaires were completed by participants (GPs) using Qualtrics, a specialist online survey software. The study was conducted between the 22 November and 11 December 2020. Once started, GP responders had a maximum of 1 month to complete the survey. All open surveys that were ongoing on 11 December 2020 were closed, and data collected up until this date were recorded. Ethical approval was granted by Loughborough University Human Participants Sub-Committee. All participants provided informed consent before completing the online survey.

### Study participants

The RCGP sent the survey link to members who had previously agreed and provided consent to be contacted about research and policy initiatives conducted by the College. Some GPs were also recruited via a link to the survey posted on social media. Only GPs working in the UK were eligible to take part. Medical students and retired GPs were excluded from the data analysis.

### Questionnaire themes

The questionnaire contained five sections, two of which were optional (this was to maximise the responses for the primary objectives). Sociodemographic data, including age category, sex, ethnicity, and job role; views about the use of sit–stand desks within general practice; and participation in physical activity^17^ were completed first. These were followed by optional questions about the number of hours worked and total time spent sitting at work each day, and items relating health and wellbeing, including height, weight, feelings of burnout,^18^ musculoskeletal health,^19^ and psychological wellbeing.^20^ GPs were only asked to reflect on using sit–stand desks in face-to-face consultations with adult patients able to stand. An open text box was also included to give GPs the opportunity to make additional comments should they wish to.

### Data analysis

Data were analysed using IBM SPSS Statistics (version 27). Data were first analysed to describe the characteristic of the recruited sample of GPs. Descriptive statistics summarised GPs’ views of the use of sit–stand desks. Analysis of covariance (ANCOVA) was used to examine whether the views of GPs differed between age categories, sex, the number of sessions worked each week, adherence to physical activity guidelines, and current use of a sit–stand desk at work (adjusted for year of qualification as GP, job role, practice list size, and ethnicity). The open text comments made by GPs were thematically coded. Data on health, wellbeing, physical activity, and sedentary behaviour are reported elsewhere.

## Results

### Response rate and participant characteristics

A total of 14 142 GPs were invited to complete the survey and 810 (5.3%) responded. Of these, 777 GPs completed the survey via the RCGP link and 33 using the social media link. Of these 777 responders, 630 contained valid data (at least 27.0% of questions answered). In total, 561 responders answered all the non-optional questions. Most responders were female (66.0%), White (78.6%), GP partners (53.3%), current members of RCGP (93.5%), and worked 3–6 clinical sessions per week (55.5%). Responses were recorded from GPs located across all regions and countries of the UK, with a reasonable balance between GPs working in cities, towns, and rural settings (Table 1).

**Table 1.** Participant characteristics (*n* = 630)

	Characteristic	*n,* (%)
**Age, years**	24–40	155 (26.1)
	41–50	237 (39.9)
	51–70	202 (34.0)
**Sex**	Male	197 (33.2)
	Female	392 (66.0)
	In another way^a^/ Prefer not to say	5 (0.8)
**Ethnicity**	White	467 (78.6)
	Other	127 (21.4)
**GP working region**	North East England	15 (2.5)
	North West England	61 (10.3)
	Yorkshire and the Humber	43 (7.2)
	West Midlands	62 (10.4)
	East Midlands	42 (7.1)
	East of England	53 (8.9)
	South West	86 (14.5)
	South East	65 (10.9)
	Greater London	59 (9.9)
	Wales	30 (5.1)
	Scotland	65 (10.9)
	Northern Ireland	13 (2.2)
**GP working location**	City	197 (33.3)
	Town	294 (49.7)
	Rural	100 (16.9)
**Job role**	Managing or executive partner	22 (3.7)
	GP partner	315 (53.3)
	Salaried GP	160 (27.1)
	Locum GP	59 (10.0)
	GP specialist	35 (5.9)
**Sessions of work per week**	1–3	65 (11.0)
	4–6	328 (55.5)
	7 or more	198 (33.5)
**RCGP Active Practice Charter**	Yes	29 (4.9)
	No	261 (44.3)
	Don’t know	299 (50.8)

RCGP = Royal College of General Practitioners.

Missing data by category: Age *n* = 36. Sex *n* = 36. Ethnicity *n* = 36. Working region *n* = 36. Working location *n* = 39. Job role *n* = 39. Sessions of work per week *n* = 39. Active practice totals *n* = 41.

^a^Any response other than ‘male’ or ‘female’.

### Views about sit–stand desks

**Figure 1.**
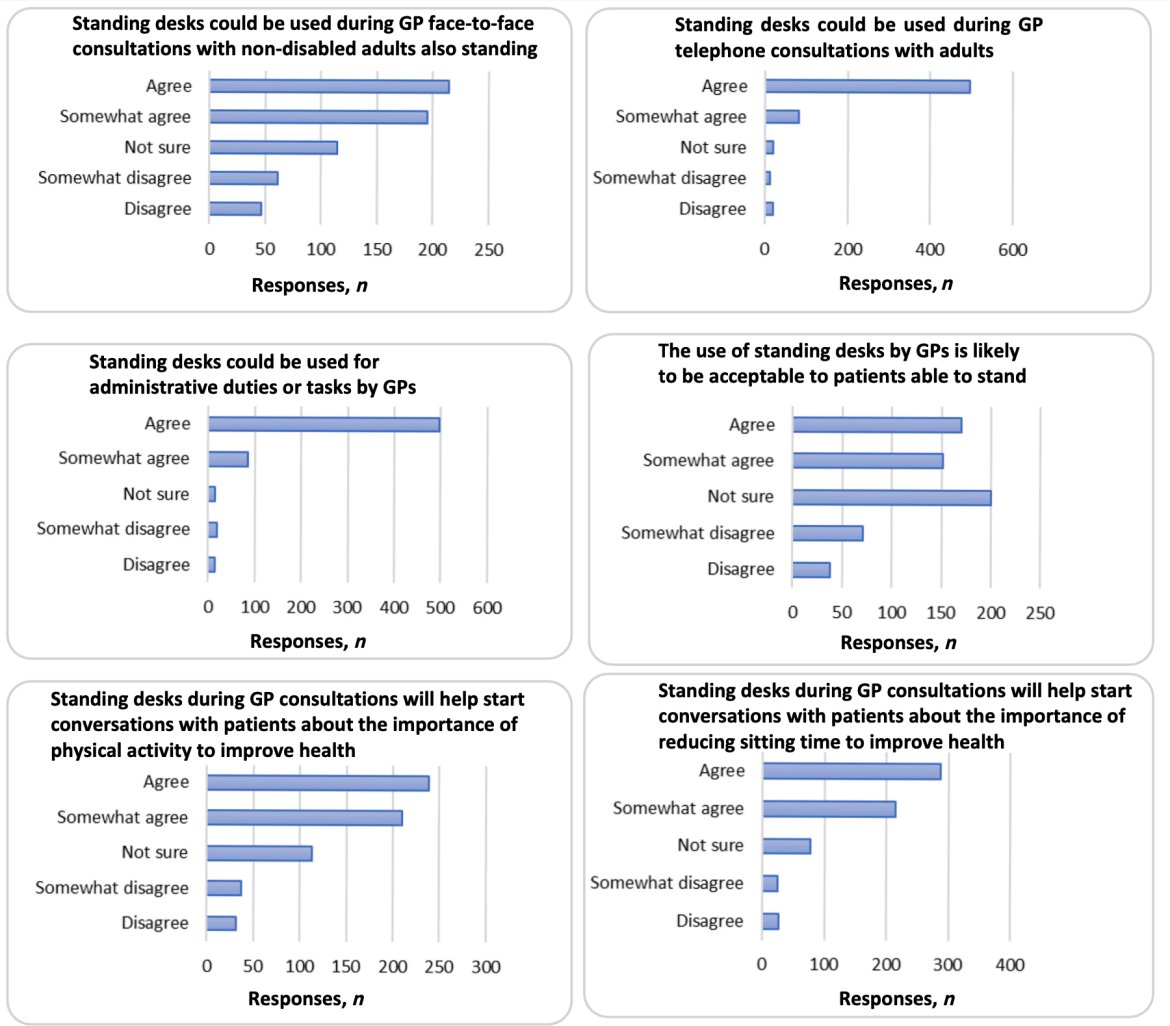
GPs’ views of the potential use of sit–stand desks. In the original survey, the first question, ‘Standing desks could be used during GP face-to-face consultations with non-disabled adults also standing’, used the term ’able-bodied’ to describe adult patients. This has been changed to ‘non-disabled’ to reflect current UK government recommendations.

Most GPs (60.6%) would like a sit–stand desk in their consultation room, while 35.0% agreed/somewhat agreed that they could be used for face-to-face consultations. Most GPs agreed sit–stand desks could be used for telephone consultations (91.9%) and administrative tasks (92.3%), but they were less certain about how acceptable they would be to patients within face-to-face consultations: 17.3% of GPs did not think that sit–stand desks would be acceptable to patients. Most GPs agreed/somewhat agreed that sit–stand desks would help start conversations with patients about the importance of reducing sitting time (79.8%) and about increasing their physical activity for optimal health (71.2%), as shown in Figure 1.

### GPs who already had a sit–stand desk

A total of 121 (19.2%) GPs already had a sit–stand desk in their consultation room, and all but one of these GPs reported that they used it at work, with most using it for telephone consultations and/or administration or meetings. Of these 121 responders, 44 (36.4%) used their sit–stand desk for face-to-face consultations with patients.

### Views about sit–stand desks according to demographic characteristics

GPs aged 41–50 years were more likely to believe that sit–stand desks would be acceptable to patients, compared to younger GPs aged 24–40 years (*P* = 0.04). Those aged 41–50 years also expressed more favourable views about the potential contribution that sit–stand desks could have on facilitating conversations with patients about their physical activity levels (*P* = 0.02) and sitting time (*P* = 0.03) compared to those aged 24–40 years. Female GPs expressed more favourable views than their male counterparts about the potential contribution that sit–stand desks could have on facilitating conversations with patients about the importance of physical activity (*P* = 0.02) and reducing sitting time (*P*≥0.01). GPs working 4–6 or 7–9 sessions per week were more positive about the potential use of sit–stand desks during face-to-face consultations (*P*≥0.01), telephone consultations (*P*≥0.01) and for administrative tasks (*P* = 0.02), compared to GPs working 1–3 sessions per week (Supplementary Table S1).

### Views about sit–stand desks according to characteristics

There was no difference in GPs views about the potential use and impact of sit–stand desks between those who met the current physical activity guidelines and those who didn’t. GPs who currently use sit–stand desks at work did think more favourably about their use during telephone consultations (*P*≥.01) and for administrative tasks (*P*≥0.01).

### Impact on the doctor–patient relationship

A total of 271 GPs (43.0%) reported being unsure of the impact that sit–stand desks would have on the doctor–patient relationship, with 26.2% expressing they thought that sit–stand desks would somewhat negatively influence the relationship. GPs were largely unsure what impact sit–stand desks would have on their ability to concentrate at work (42.2%), patients’ perceptions of their listening skills (51.3%), and patients’ understanding of issues discussed in the consultation (68.6%). However, only a small percentage of GPs thought sit–stand desk would negatively impact their ability to concentrate (4.3%), patients’ perceptions of their listening skills (8.4%), and patients’ understanding of issues discussed in the consultation (4.0%). Those aged ≥41 years were more positive than those aged 24–40 years about the potential impact that sit–stand desks would have on GPs’ concentration (*P* = 0.03), patients' perception of GPs listening skills (*P*≥0.01), and patients’ understanding of issues discussed in the consultation (*P* = 0.02); see Supplementary Table 2.

### Barriers to the implementation of sit–stand desks

Most GPs felt there was the potential for issues with doctor–patient communication arising from the use of a sit–stand desk in face-to-face consultations, which was thought to be the most likely barrier to successful implementation. About half (51.9%) of GPs reported environmental or organisational issues as potential barriers; for example, lack of financial resource and workload pressures. Only 21.4% of GPs reported personal issues as potential barriers to implementation; for example, lack of time to stand or perception that it is uncomfortable to do so.

### GPs’ health and sit–stand desks

Most GPs believed sit–stand desks would reduce their sitting time (94.6%); increase their physical activity (89.8%); and improve their posture or musculoskeletal health (88.1%), wellbeing (79.0%), and feelings of fatigue at the end of the working day (54.6%), as shown in Table 2. About two-thirds of responders (62.0%) met the World Health Organization’s aerobic physical activity guidelines of achieving participation in ≥150 minutes of moderate intensity physical activity per week, while only 37.2% met the muscle strengthening guidelines of achieving muscle strengthening activity ≥2 days per week. When combined, only 29.4% of GPs met both guidelines. Most of the time at work was spent sitting (73.0%), with 13.6% of sitting time spent in prolonged bouts of sitting (≥30 minutes). Half of GPs reported having one or fewer breaks in sitting per hour (such as standing up, stretching, or taking a short walk).

**Table 2.** GPs’ views of the potential impact of sit–stand desks on their physical behaviour, health, and wellbeing

	Perception	Task		
		Face-to-face consultation, *n* (%)	Telephone consultation, *n* (%)	Administration , *n* (%)
**Sitting time**	Negatively/somewhat negatively	15 (2.4)	19 (3.0)	23 (3.7)
	Unsure	19 (3.0)	19 (3.0)	23 (3.7)
	Positively/somewhat positively	596 (94.6)	592 (93.9)	584 (92.7)
**Physical activity**	Negatively/somewhat negatively	17 (2.7)	14 (2.2)	16 (2.5)
	Unsure	47 (7.5)	37 (5.9)	45 (7.1)
	Positively/somewhat positively	566 (89.8)	579 (91.9)	569 (90.3)
**Posture**	Negatively/somewhat negatively	23 (3.7)	25 (4.0)	29 (4.6)
	Unsure	52 (8.3)	48 (7.6)	50 (7.9)
	Positively/somewhat positively	555 (88.1)	557 (88.4)	551 (87.4)
**Musculoskeletal health**	Negatively somewhat negatively	23 (3.7)	21 (3.3)	28 (4.4)
	Unsure	52 (8.3)	51 (8.1)	50 (7.9)
	Positively/somewhat positively	555 (88.1)	558 (88.6)	552 (87.6)
**Wellbeing**	Negatively somewhat negatively	25 (4.0)	29 (4.6)	32 (5.1)
	Unsure	107 (17.0)	84 (13.3)	90 (14.3)
	Positively/somewhat positively	498 (79.0)	517 (82.1)	508 (80.6)
**Fatigue at the end of the day**	Negatively/somewhat negatively	90 (14.3)	83 (13.1)	81 (12.9)
	Unsure	196 (31.1)	175 (27.8)	176 (27.9)
	Positively/somewhat positively	344 (54.6)	372 (59.0)	373 (59.2)

### Themes from open text comments

Of the 630 GPs to provide valid data, 212 left open text comments relating to the potential use of sit–stand desks within general practice. Key themes to emerge from these responses were cost (13 comments), potential impact on the doctor–patient relationship (nine positive comments and 34 negative comments), impact on health and wellbeing of GPs (26 positive comments and three negative comments), the impact on the energy and concentration of GPs (10 positive comments and one negative comment), and the potential positive influence on patients’ health-related behaviours (six comments).

## Discussion

### Summary

This study showed that the implementation of sit–stand desks within general practice would have support from GPs, who believe there would be benefits from doing so. The findings highlighted that sit–stand desks were considered appropriate by GPs for most work-related tasks, although there was not a clear consensus about whether they could be used during face-to-face patient consultations, with 43.0% of GPs unsure of the potential impact of the desks on the doctor–patient relationship. Most GPs thought that sit–stand desks would help start conversations with their patients about the importance of reducing their sitting time and participation in physical activity for maintaining good health. GPs reported sitting for about three-quarters of their working day and few (29.4%) reported participating in sufficient physical activity for optimal health.

### Strengths and limitations

This study has several strengths, including a large sample size with responses from GPs working across all countries of the UK. To the authors' knowledge, this was the first study to investigate the views of GPs on the question of whether sit–stand desks should be implemented in general practice. This study also has some limitations. The response to the survey was modest (5.3% from the RCGP link) and those who responded may have held more favourable views than GPs who did not respond, although descriptive data shows that those who did respond varied according to sex, ethnicity, and region of the UK, providing some reassurance the findings represent a broad range of GPs. The cross-sectional nature of findings may limit the wider applicability of sit–stand desks by GPs, but the findings of this study can be used to inform the design of future research endeavours on this question and the working practices of GPs. It is also important to note that working hours only form part of a GP's day, therefore time outside this (leisure time), and levels of physical activity and sedentary behaviour during this time should be examined in future research.

### Comparison with existing literature

Although this study is the first to investigate GPs views about the use of sit–stand desks during their working day, the findings are consistent with evidence showing that sit–stand desks are widely acceptable to office workers.^13,21,22^ Given that previous evidence suggests that secondary care patients’ views of their doctor can vary depending on whether they are sitting or standing while delivering care or during consultations as part of hospital inpatient stays,^23^ it is equally important that patients’ views about their GP using sit–stand desk during face-to-face consultations are explored in future research. Such research may help to identify strategies to mitigate any potential negative impact on the patient consultation experience and guide future policy.

### Implications for research and practice

This study has highlighted that there is a willingness by GPs to use sit–stand desks within patient consultations and for other work tasks. However, it would be premature to proceed with a widescale implementation of sit–stand desks in general practice before evidence about their impact on the working day and health of GPs is known. Data on the views and experiences of patients from participating in GP standing consultations are also imperative. In future research, it would also be important to assess whether standing consultations lead to GPs and patients sitting less and being more physically active. As highlighted earlier, the findings regarding the use of sit–stand desks during face-to-face consultations was mixed. Future research should consider investigating this specific question in more detail to explore what the concerns might be for GPs. There are already strategies in primary care that are designed to promote physical activity in patients, such as the Active Practice Charter, and sit–stand desks could be couched within such campaigns as a further method of promoting physical activity with patients.

In conclusion, GPs in the UK generally supported the implementation of sit–stand desks within general practice. Future use of sit–stand desks during face-to-face consultations should be carefully managed, with consideration given to the potential impact on the doctor–patient relationship. Any future research involving sit–stand desk interventions with GPs should focus on understanding the views of patients and whether such a change to their consultations with the GP is acceptable.
